# Auxin regulates first leaf development and promotes the formation of protocorm trichomes and rhizome-like structures in developing seedlings of *Spathoglottis plicata* (Orchidaceae)

**DOI:** 10.1093/aobpla/pls053

**Published:** 2013-01-08

**Authors:** Stacey D. Novak, Grace A. Whitehouse

**Affiliations:** 1 Department of Biology, University of La Verne, 1950 3rd Street, La Verne, CA 91750, USA; 2 Indiana University, School of Public Health, 1025 E. Seventh Street, Bloomington, IN 47405, USA

**Keywords:** Auxin, PAT inhibitors, propagation, protocorm trichomes, rhizome-like structures, seedling development, *Spathoglottis*.

## Abstract

Auxin flows in a polar manner to target tissues and exert its morphogenic effect. Preventing auxin movement, with polar auxin transport (PAT) inhibitors, or increasing auxin levels in tissues through exogenous application can provide a means for assessing the importance of appropriate tissue distribution and concentration of this hormone during development. The formulation of culture media for micropropagation has been the primary focus of most orchid tissue culture research, a goal that unveils seedling hormone responses at a single point in development. This study was unique because it evaluated the auxin response of orchids during three stages of seedling development. Seedlings were grown on standard culture media for 10, 35 and 85 days. Each group was sub-cultured onto auxin- and/or PAT inhibitor-containing media for an additional 10, 30 and 60 days, respectively. Data were collected on first leaf initiation, trichome formation and the appearance of propagative structures. In the 20-day seedlings, auxins and PAT inhibitors promoted precocious formation and random placement of protocorm hairs rather than in tufts, as seen in older, control seedlings. The 65-day seedlings formed protocorm-like bodies, rhizome-like growths from the stem, and fleshy leaves with trichomes. Seedlings cultured for 145 days developed microshoots or callus growth in the axils of older leaves and exhibited necrosis of original seedling roots and leaves. In general, exogenously applied auxin promoted the reversion of differentiated *Spathoglottis plicata* seedling tissue to a morphology that had propagative properties. Additionally, auxins commonly induced hair formation, which suggests that protocorm hairs may be root hair-like in nature. This work characterized three auxin growth responses in *S. plicata* seedlings that have not been reported in orchids: (i) the inhibition of first leaf initiation and abnormal first leaf morphology; (ii) the promotion of trichome formation; and (iii) the induction of rhizome-like structures and microshoots at a specific stage in seedling development.

## Abstract (precis)

This work demonstrated that auxin plays an important role in seedling establishment of the orchid *S. plicata*. In particular, normal first leaf formation required an appropriate distribution and concentration of auxin, as revealed by the use of polar auxin transport inhibitors and exogenously applied auxin. Moreover, auxin promoted the precocious formation of protocorm trichomes, which are an essential part of normal orchid seedling development. In addition, this work revealed that auxin can induce orchid seedlings to form propagative structures that are specific to a particular stage in seedling development.

## Introduction

Phytohormones are key ingredients in the design of orchid culture media due to their effect on tissue and organ morphogenesis. Many of these hormone-containing recipes induce the formation of propagative structures, such as protocorm-like bodies (PLBs), rhizomes, callus tissue and microshoots (Arditti 2008). Micropropagation, through the use of explants, has been a primary emphasis for most orchid researchers, since it addresses the rapid cultivation needs presented by threatened or endangered species, the increasing sales of ornamental plants, and the demands of the cut flower industry (Hew and Yong 2004; Swarts and Dixon 2009). However, studies with tissue or organ explants, rather than whole seedling explants, reveal very limited information about the importance of hormone concentrations in seedling development. Understanding hormonal impacts on seedling morphogenesis will provide insights about development and enable researchers and growers to be more prescriptive in media design for future studies and cultivation. In this work, the impact of auxin on seedling development was studied in *Spathoglottis plicata*, a terrestrial orchid species that is sold commercially by many growers. Specifically, this work addressed the impact of auxin distribution on the initiation of first leaves and the effect of exogenous auxin application on the formation of trichomes and propagative structures in *S. plicata* seedlings at three stages of development.

The unique ability of auxin to move in a polar fashion allows for differential tissue distribution, which is a key factor in aspects of plant morphogenesis (Vanneste and Friml 2009). For example, during *Arabidopsis* embryogenesis, auxin migrates to two distal regions of the globular embryo for the induction of cotyledon development (Jenik and Barton 2005). Further, localized indole-3-acetic acid (IAA) dictates the radial position and size of a leaf during organ formation in the *Arabidopsis* shoot (Reinhardt *et al.* 2000). Polar auxin transport (PAT) requires pin-formed (PIN) proteins that function in the export of auxin from the cell (Friml and Palme 2002). Pin-formed protein is delivered to the membrane via endosome action, but basipetal or acropetal movement is accomplished through selective endocytic removal of PIN from the membrane (Baluska *et al.* 2008; Dhonukshe *et al.* 2010). Auxins and PAT inhibitors, such as monensin, prevent the removal of PIN proteins from appropriate regions of the plasma membrane, thus interrupting the polar flow of auxin to the site of action (Paciorek *et al.* 2005; Dhonukshe *et al.* 2008). Through the use of PAT inhibitors, Reinhardt *et al.* (2000) and Scanlon (2003) have shown that auxin is necessary for leaf initiation in tomato and maize apices. In addition to leaves, auxins have been directly implicated in the formation of lateral roots and floral organs (Vanneste and Friml 2009). In orchids, auxin induces the formation of microshoots from nodal segments and rhizomes from pseudobulb tissue (Shimasaki and Uemoto 1990; Sinha *et al.* 2009).

Unlike seeds of typical angiosperms, most mature orchid seeds are a small group of undifferentiated cells that lack a primordial shoot/root axis. In some genera, such as *Cymbidium* and *Spathoglottis*, a large suspensor cell denotes the distal end, while the proximal end is comprised of considerably smaller cells (Raghavan and Goh 1994; Vinogradova and Andronova 2002; Yam *et al.* 2002). Even though the embryonic axis polarity is not clearly defined, in *S. plicata* the shoot always arises from the proximal end (Raghavan and Goh 1994; Novak *et al.* 2008). Under culture conditions, germination of this species begins with the formation of a ball of undifferentiated cells called the protocorm. Approximately 12–14 days after culture (DAC), this structure becomes a seedling, as it initiates a predominant first leaf with a smaller second leaf coiled inside. By 35–40 DAC, seedlings have developed young leaves and protocorm hairs have formed in distinctive groupings or ‘tufts’ on the distal end of the seedling. Roots begin to emerge at 65–70 DAC (Prakash and Aow 1973; Raghavan and Goh 1994; Novak *et al.* 2008).

Trichomes, which play an important role in plant survival, also respond to hormone application. Hairs found on leaves and stems have numerous functions, such as secretion and light reflection. These trichomes increase in number in response to jasmonic acid and gibberellic acid (GA), but they are either non-responsive to exogenous auxin application or revert to glandular-like hairs (Traw and Bergelson 2003; Kim *et al.* 2007). In contrast, root hairs serve in water/nutrient uptake and their growth is promoted by ethylene and auxin (Rahman *et al.* 2002; Ishida *et al.* 2008). Protocorms of all orchid species produce hairs. However, the nature of these hairs has not been closely studied.

Since auxins play an important role in plant morphogenesis, the goal of this study was to explore their impact on orchid seedling development. Researchers have reported different responses of orchid seedlings to synthetics when compared with natural hormones (Sharma and Tandon 1986; Park *et al.* 2003). Therefore, in order to make this a thorough study, both IAA and 2-4-dichlorophenoxyacetic acid (2,4-D) were tested.

In this work, *S. plicata* seedlings were exposed to PAT inhibitors and auxins at 10 DAC, and auxins at 35 and 85 DAC. One objective of this work was to use the PAT inhibitors, *N*-1-naphthylphthalamic acid (NPA), 2,3,5-triiodobenzoic acid (TIBA) and monensin, in addition to auxins (IAA and 2,4-D), to evaluate the role of auxin distribution in normal first leaf formation. A second aim of this work was to better characterize the nature of protocorm hairs by studying whether exogenously applied auxins enhanced their formation. The third directive was to determine how auxin application would impact organ morphogenesis during *S. plicata* seedling development, and if this response was developmental stage specific.

## Materials and methods

### Seed culture and seedling sub-culture

*Spathoglottis plicata* pods were collected from wild plants in Oahu, Hawaii. Green pods were surface sterilized by dipping in alcohol and flaming. Twelve pods were allowed to dehisce naturally and seeds were removed for surface sterilization by gently tapping the dehisced pod in a sterile Petri dish. About 15.0 mg of dry seeds were placed in a 1.5 mL microfuge tube, briefly rinsed in 70 % ethanol (v/v) and washed in sterile demineralized water (DMW) before being treated with 0.2 % NaOCl (v/v). After 1 min in bleach, seeds were washed in six rinses of sterile DMW. The NaOCl solution was freshly prepared from Clorox bleach with a NaOCl concentration of 6 % and an available chlorine level of 5.7 %.

Seeds were cultured on standard media, Phytamax Orchid Maintenance Media (Sigma-Aldrich Chemical, St Louis, MO, USA) with 0.7 % (w/v) agar. Media were adjusted to a pH of 6.5 and autoclaved. All growth regulators and PAT inhibitor stock solutions were added to the freshly autoclaved medium after it had been cooled to 50 °C. Approximately 200 seeds were initially sown on each of 120 replicate plates in preparation for sub-culturing. Seedlings were grown on standard media until they reached the appropriate stage for sub-culture (10, 35 or 85 DAC) and then cultured on PAT inhibitor or auxin-containing media until responses were evident in the seedlings (20, 65 and 145 DAC, respectively). Sub-culturing was done on 10 replicate plates for each treatment for the 10 DAC seedlings, and on five replicate plates for the 35 and 85 DAC seedlings. Seedling groups from the initial plates were divided to provide plenty of sub-culture replicate plates. For all experiments, seedlings were cultured in a plant growth chamber at 25 °C on an 8/16 h light/dark cycle under cool white lights (Lab-Line Instruments, Melrose Park, IL, USA).

After 10 days in standard culture, seedlings had reached a protocorm stage just prior to first leaf initiation. Protocorms were then sub-cultured on standard media supplemented with PAT inhibitors or auxins. A natural auxin (IAA) and a synthetic auxin (2,4-D) were used to verify that the responses were not unique to natural auxins. 2,3,5-Triiodobenzoic acid (Sigma-Aldrich Chemical) was added to final concentrations of 10 and 100 µM, monensin (MP Biomedicals LLC, Solon, OH, USA) to concentrations of 50 and 100 µM, NPA (Supelco, Bellefonte, PA, USA) to concentrations of 30 and 100 μM, IAA (Sigma-Aldrich Chemical) to concentrations of 0.75 and 1.5 μM, and 2,4-D (Sigma-Aldrich Chemical) to concentrations of 1 and 2 μM. After 10 days in culture, data were collected. A second group of seedlings was sub-cultured on standard media supplemented with auxin after 35 days in standard culture. At this stage, seedlings had developed leaves, and protocorm hairs had begun to form. The auxin, 2,4-D, was added to standard media at a final concentration of 2 μM and IAA to a concentration of 0.75 μM. After 30 days in culture, data were collected. A third group of seedlings was sub-cultured on standard media supplemented with auxin after 85 days in standard culture. At this stage, seedlings were well established with developed roots. The auxins, 2,4-D and IAA, were added to standard media at a final concentration of 0.75 μM. After 60 days in culture, data were collected.

### Microscopy

Images of wet mounts, sectioned material and whole seedlings were captured using a digital compound microscope, a digital dissecting microscope and a scanning electron microscope (SEM). For sectioned material, rhizome-like structures were dehydrated in an ethanol series and embedded in a glycol methacrylate plastic embedding medium, JB-4 (Polysciences, Warrington, PA, USA). Methacrylate blocks were sectioned on an MT-990 microtome (Boeckeler Instruments, Tucson, AZ, USA). Sections were stained with 0.05 % toluidine blue O in benzoate buffer pH 4.4 for 3 min and rinsed in DMW for 1 min. For the SEM image, a small representative section was taken from an IAA-treated, 145-day seedling and placed on a sample stub. It was directly photographed under the SEM.

### Data collection and statistics

For the 20 DAC seedlings, random samples of 200 seedlings were collected from the replicate plates for each treatment. Data were taken on the following growth parameters: protocorm diameter, percentage of seedlings with hairs, percentage of seedlings with a first leaf, and first leaf length. However, the 2,4-D treatment had very low first leaf initiation and, as a result, only 62 and 10 seedlings were used for the 1 and 2 μM treatments, respectively. Percentage data were calculated from four groups of 50 seedlings. For the 65-day seedlings, data were taken from 60 seedlings for the following parameters: percentage of seedlings with callus tissue, hairs on leaves and hairs on rhizome-like structures, and the number of PLBs/rhizome-like growths. Percentage data were calculated from three groups of 20 seedlings. Spot Advanced Software (Diagnostic Instruments, Sterling Heights, MI, USA) was used to determine first leaf lengths and protocorm diameters. Data means were subjected to analysis of variance and separation was achieved by Duncan's multiple range test (DMRT).

## Results

### Polar auxin transport inhibitors and exogenously applied auxins inhibit first leaf formation, promote hair growth and increase protocorm diameters in young seedlings (20 DAC)

**Fig. 1:**
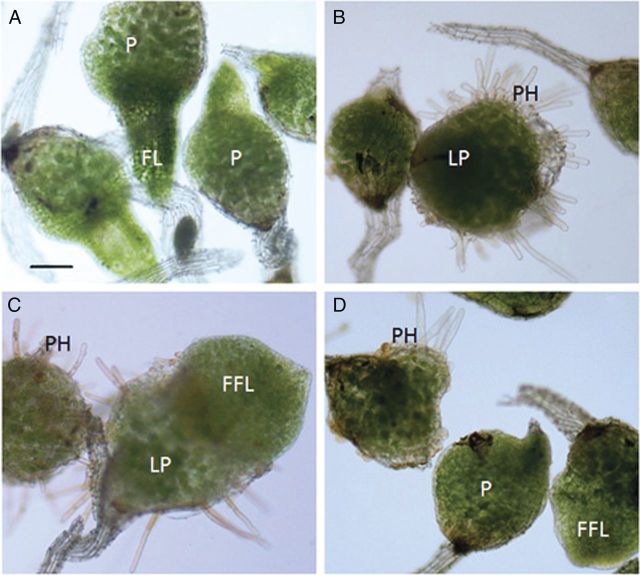
**Effects of auxin and PAT inhibitors on 10 DAC seedlings.** Seedlings were grown for 10 days on standard media and then sub-cultured onto standard media (control), auxin- or PAT inhibitor-containing media for an additional 10 days. (A) Control seedlings with protocorm (P) and first leaf (FL) formation. (B–D) Seedlings sub-cultured on media with 1.5 μM IAA, 2 μM 2,4-D, 100 μM TIBA, respectively, produced protocorm hairs (PH), large protocorms (LP) and fleshy first leaves (FFL). Bar = 150 μm.

Protocorms grown from seed sown on standard media for 10 days were sub-cultured onto media that had been supplemented with PAT inhibitors or auxin, and seedlings were grown for an additional 10 days. In contrast to the control seedlings, auxin- and PAT inhibitor-treated seedlings had protocorms that either lacked a first leaf or generated short, fleshy leaves (Fig. 1). Of all treatments, those exposed to 2 μM 2,4-D were most affected, having only 5 % first leaf emergence compared with 80 % in the control (Table 1). 3-Indoleacetic acid-treated protocorms had first leaf percentages comparable to those of seedlings grown on 100 μM monensin or TIBA, ∼40 % (Fig. 1B, D; Table 1). 2,3,5-Triiodobenzoic acid and monensin exhibited a dosage-dependent response, such that higher levels more effectively prevented first leaf formation or stunted first leaf growth (Table 1; Fig. 1D). *N*-1-Naphthylphthalamic acid was not effective in preventing first leaf formation, since it had the same percentage of seedlings with a first leaf as the control group. However, it had significantly shorter leaves than the control, similar to what was observed with TIBA- or monensin-treated seedlings (Table 1; Fig. 1A and D). Moreover, the leaves tended to fuse together, creating stunted leaves with a cleft to separate them. This was most visible in the monensin-treated seedlings.

**Table 1:** **Effects of auxin and PAT inhibitors on the morphology of 20 DAC seedlings**. Seed was sown on standard media to produce 10-day seedlings. These seedlings were then sub-cultured onto auxin (IAA or 2-4,D) or PAT inhibitor-containing (monensin, TIBA or NPA) media and grown for an additional 10 days. Measurements of the first leaf and protocorm were made using Spot Advanced software. Values are an average of data taken from 200 seedlings. Percentage data were collected from four groups of individual experiments. All values are means with standard errors. Measurements with the same letter for a given parameter are not significantly different at *α* = 0.05 (DMRT).

Treatment (μM)	Protocorm diameter (μm)	First leaf length (μm)	% Seedlings with leaf emergence	% Seedlings with protocorm hairs
Control: before sub-culture	264.39 ± 2.53^a^	NA	4.53 ± 2.49^a^	NA
Control: after sub-culture	346.72 ± 4.34^b^	188.12 ± 7.65^a^	79.13 ± 2.11^ef^	1.45 ± 0.93^a^
Monensin (50)	346.93 ± 4.44^bc^	102.30 ± 4.16^de^	66.50 ± 2.50^e^	6.72 ± 0.49^ab^
Monensin (100)	350.42 ± 4.40^bcd^	81.09 ± 3.96^e^	43.00 ± 6.65^cd^	12.95 ± 2.14^bc^
TIBA (10)	363.84 ± 4.28^cde^	137.92 ± 5.71^bc^	73.00 ± 3.785^ef^	8.30 ± 2.84^ab^
TIBA (100)	359.25 ± 3.86^bcd^	105.30 ± 4.32^de^	38.50 ± 0.95^bc^	17.75 ± 1.03^c^
NPA (30)	372.06 ± 3.84^ef^	118.89 ± 3.78^cd^	83.50 ± 4.27^g^	0.40 ± 0.03^a^
NPA (100)	369.60 ± 3.77^cde^	113.49 ± 3.53^cd^	75.00 ± 3.69^ef^	0.90 ± 0.43^a^
IAA (0.75)	414.56 ± 4.45^f^	100.15 ± 4.21^de^	44.00 ± 11.43^cd^	44.15 ± 1.82^e^
IAA (1.5)	380.51 ± 4.34^cde^	84.22 ± 4.47^e^	52.00 ± 5.03^c^	27.45 ± 2.53^d^
2-4,D (1)	464.23 ± 5.41^g^	149.16 ± 8.89^b^	29.00 ± 5.91^b^	54.50 ± 5.73^f^
2-4,D (2)	488.28 ± 5.16^h^	106.20 ± 11.64^de^	4.50 ± 2.52^a^	80.75 ± 4.42^g^

Protocorm hair formation in the control seedlings occurred at the distal end in 2 % of the seedlings (Fig. 1A; Table 1). Hair formation was moderately increased with the PAT inhibitors, monensin and TIBA, but auxins caused a large increase in the production of protocorm hairs (Fig. 1B–D; Table 1). Monensin and TIBA exhibited a dosage response as the PAT inhibitor concentration increased from 0 to 100 μM, with the 100 μM treatment causing hair formation in ∼15 % of the seedlings. 3-Indoleacetic acid at a concentration of 0.75 μM and 2,4-D at a concentration of 2 μM stimulated 40 and 80 % of the seedlings, respectively, to generate protocorm hairs (Table 1).

Protocorm diameters increased when exposed to auxins, but those in the PAT inhibitors, TIBA and NPA, were not larger than the control (Fig. 1A–D; Table 1). When comparing the control before sub-culture with the control after sub-culture, the protocorm increased in diameter. While the NPA-treated seedlings had slightly larger protocorms than the control, IAA and 2,4-D treatments produced seedlings with protocorm diameters that were 15 and 30 % larger, respectively, than the after-sub-culture control (Table 1).

### Exogenously applied auxin promotes the formation of PLBs/rhizome-like structures, callus tissue, microshoots and leaf hairs in older seedlings (65 and 145 DAC)

**Fig. 2:**
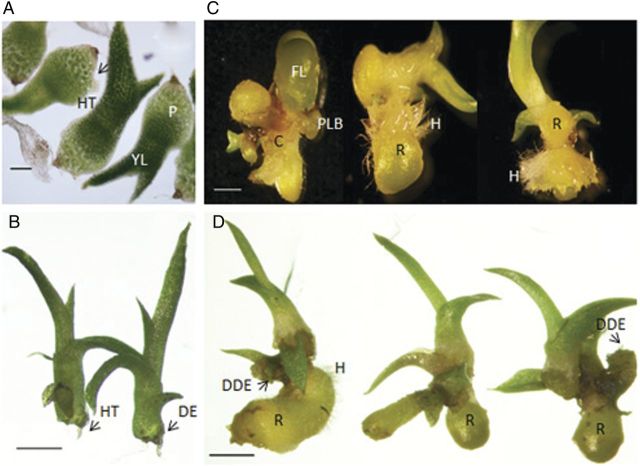
**Effects of auxin on 35 DAC seedlings.** Seedlings were grown for 35 days on standard media and then sub-cultured onto standard media (control) or auxins for an additional 30 days. (A) Seedlings before sub-culture (35 DAC) had protocorms (P), hair tufts (HT) and young leaves (YL). Bar = 200 μm. (B) Control seedlings after the 30-day sub-culture on standard media had well-developed leaves and hair tufts emerging from the distal end (DE) of the residual protocorm. Bar = 1 mm. (C) Seedlings after a 30-day sub-culture on 2,4-D media had protocorm-like bodies (PLB), rhizome-like structures (R), callus (C), fleshy leaves (FL) and hairs (H). Bar = 1 mm. (D) Seedlings after the 30-day sub-culture on IAA had a displaced distal end (DDE), rhizome-like structures and hairs. Bar = 2 mm.

**Fig. 3:**
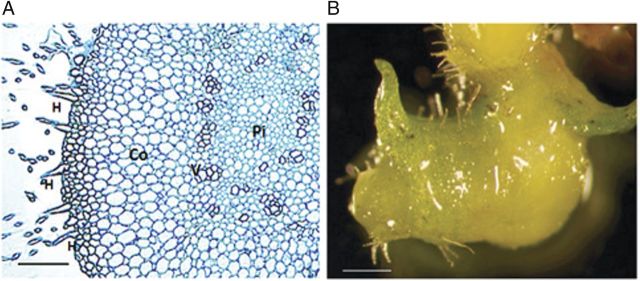
**Hair formation in 65 DAC seedlings.** Hairs formed on the rhizome-like structures and leaves of those seedlings cultured on auxin-containing media. (A) Cross-section through the rhizome-like structure seen in Fig. 2C and D showing siphonostele anatomy with a pith (Pi) surrounded by vascular tissue (V), a cortex (Co) and randomly distributed hairs (H). Bar = 200 μm. (B) Hairs formed on the fleshy leaves of 2,4-D-treated seedlings. Bar = 1 mm.

Control seedlings, which were sub-cultured at 35 days and grown until 65 days on standard media, developed normal seedling leaves and stems, and hair tufts formed from the residual protocorm on the distal end of the seedling (Fig. 2A and B). Seedlings sub-cultured onto auxin-containing media at 35 DAC and grown for an additional 30 days formed growths that resembled PLBs or appeared elongated and rhizome-like (Fig. 2C and D). These rhizome-like structures formed from the stem, with many emerging from the axil of older leaves. A comparison of the control seedling morphology with that of those grown on auxin revealed that this new growth displaced the distal end of the seedling, where the hairs normally form in distinctive tufts (Fig. 2B and D). Light microscopy of transverse sections through these rhizome-like structures showed a pith and cortex tissue arrangement that is typical of rhizome anatomy (Fig. 3A).

Both IAA and 2,4-D produced more PLB/rhizome-like structures per seedling than the control, but higher numbers were seen in the 2,4-D-treated seedlings when compared with the IAA-treated ones (Table 2; Fig. 2). Callus tissue formation was observed in seedlings with both auxin treatments; however, the 2,4-D treatment had a higher percentage of seedlings with callus tissue than the IAA-containing media. Leaf hairs formed on ∼18 % of those seedlings cultured on IAA or 2,4-D, but were absent on the leaves of control seedlings (Table 2). In all cases, hairs were only observed on fused, fleshy leaves (Fig. 3B). Hairs also formed on the rhizome-like structures (Fig. 2C, D and 3A). Their distribution was uniform rather than the grouped hairs or ‘tufts’ at the distal end of the seedling stem, as seen in the control (Fig. 2A and B).

**Table 2:** **Effects of auxin on the morphology of 65-day seedlings.** Seed was sown on standard media to produce 35-day seedlings. These seedlings were then sub-cultured onto auxin (IAA or 2-4,D)-containing media and grown for an additional 30 days. Values are an average of data taken from 60 seedlings. Percentage data were collected from three groups of individual experiments. All values are means with standard errors. Measurements with the same letter for a given parameter are not significantly different at *α* = 0.05 (DMRT).

Treatment (μM)	Rhizome/PLB (no./seedling)	% Seedlings with rhizome hairs	% Seedlings with callus tissue	% Seedlings with leaf hairs
Control	0.00^a^	0.00^a^	0.00^a^	0.00^a^
IAA (0.75)	1.27 ± 0.67^b^	53.33 ± 4.41^b^	30.00 ± 2.89^b^	20.00 ± 5.77^b^
2-4,D (2)	1.98 ± 0.18^c^	73.33 ± 6.01^c^	51.67 ± 3.33^c^	16.67 ± 1.67^b^

**Fig. 4:**
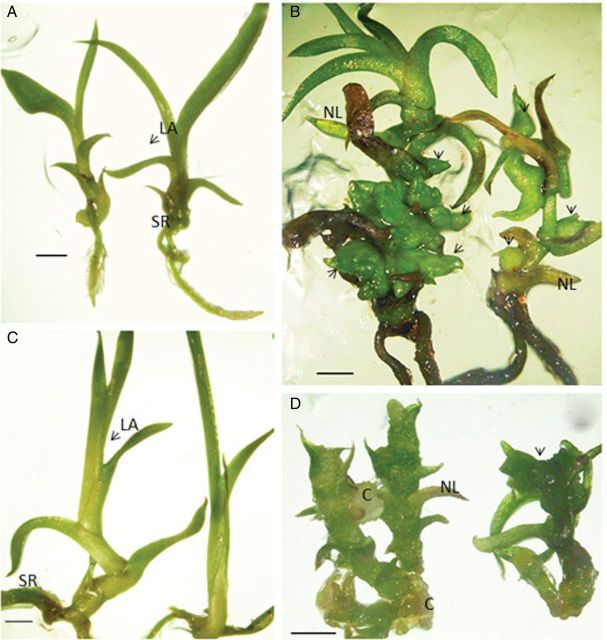
**Effects of auxin on 85 DAC seedlings.** Seedlings were grown for 85 days on standard media and then sub-cultured onto standard media (control) or auxins for an additional 60 days. (A) Control seedlings before sub-culture (85 DAC), leaf axil (LA) and seedling root (SR). (B–D) Seedlings sub-cultured at 85 DAC on standard media or auxin-containing media for an additional 60 days. (B) Control seedlings after sub-culture (145 DAC). (C) After a 60-day sub-culture on IAA-containing media, seedlings had necrotic roots (NR) and buds (at arrows) in the axils of necrotic leaves (NL). (D) After a 60-day sub-culture on 2,4-D-containing media, seedlings had callus (C) and buds. (A–D) Bar = 2 mm.

**Fig. 5:**
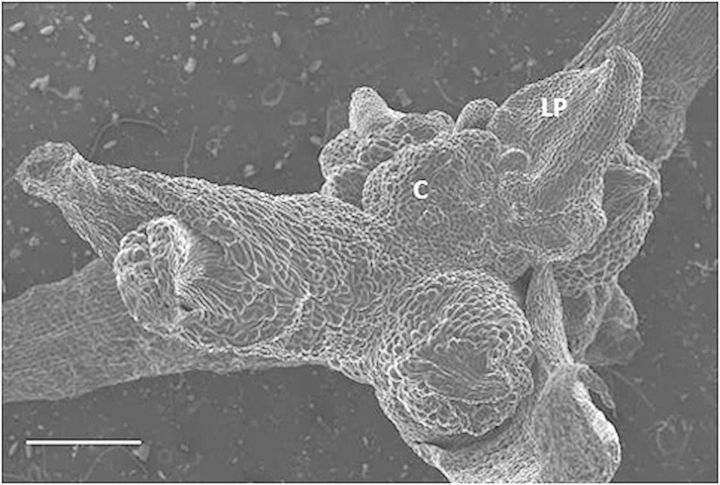
**IAA-induced callus tissue generated a new leaf.** Seedlings were grown for 85 days on standard media and then sub-cultured onto IAA-containing media for an additional 60 days. The SEM shows callus tissue (C) producing a leaf primordium (LP) in the axil of an older, necrotic leaf. Bar = 500 μm.

Control seedlings before (85 days) and after (145 days) the 60 day sub-culture had typical seedling leaves and roots (Fig. 4A and C). In contrast, seedlings sub-cultured at 85 DAC for 60 days on auxin-containing media generated buds (microshoots) or callus tissue in the axil of older leaves (Fig. 4B and D). Moreover, original roots and seedling leaves became necrotic while the new growths predominated in these seedlings (Fig. 4B and D). The IAA- and 2,4-D-treated seedlings produced single or multiple microshoots (buds) on the stem and in the axils of senescing leaves (Fig. 4B and D). The SEM shows a new shoot that arose from callus tissue in the axil of a senescing leaf on an IAA-treated seedling (Fig. 5).

### Three auxin responses were unique for a particular stage in development while others were observed during more than one stage

Inhibition of first leaf formation was only observed in 20 DAC seedlings. Rhizome and PLB formation was specific to the 35 DAC seedlings and microshoot formation only occurred in 145 DAC seedlings (Table 3). Other auxin responses exhibited overlap between two adjacent time points in development, such as trichome formation and fleshy leaves in 20 and 65 DAC seedlings or leaf necrosis and callus tissue in 65 and 145 DAC seedlings (Table 3). Four different types of propagative structure were generated, but all were formed in the older seedlings, 65 and 145 DAC. There were no cases where all developmental stages studied exhibited a common auxin response (Table 3).

**Table 3:** **Summary of seedling growth responses to auxins at three points in development.** Seedlings were sub-cultured after 10, 35 or 85 days on standard media and grown on IAA- or 2-4,D-containing media for 10, 30 or 60 days, respectively. The appearance of trichomes, propagative structures, necrotic leaves, enlarged protocorms and fleshy leaves was compared for each time point studied. An ‘X’ indicates that the given parameters are present in the seedlings.

Time in standard culture media (days)	10	35	85
Time sub-cultured on auxin (days)	10	30	60
Total culture time (days)	20	65	145
Increased protocorm diameter	X		
Inhibition of first leaf	X		
Fleshy leaves	X	X	
Trichomes	X	X	
Callus (propagation)		X	X
Microshoots (propagation)			X
PLBs (propagation)		X	
Rhizomes (propagation)		X	
Necrosis of leaves		X	X

## Discussion

The effect of auxins on plant development has led many researchers to characterize this hormone as a ‘plant morphogen’. Polar auxin transport via PIN proteins directs auxin to a specific location in the plant body during development and plays a central role in tropic growth, apical dominance, lateral root initiation, vascular development and embryo patterning (Vanneste and Friml 2009). In studies with orchids, efforts have been made to identify the optimal auxin concentration in media that is needed for propagation of a specific species, but few have focused on the role of auxin in seedling development. The objective of this study was to evaluate the impact of disrupted auxin gradients and increased concentrations on seedling morphogenesis of *S. plicata*. This was accomplished through the direct application of auxin and/or PAT inhibitors at three stages of seedling development.

In this work, the application of auxins or PAT inhibitors early in development (10 DAC seedlings) resulted in the suppression of first leaf emergence or the development of thick, stunted, fleshy leaves. This common response suggests that auxin polarity plays an important role in first leaf initiation and leaf morphogenesis. These results are consistent with studies in *Arabidopsis* embryogenesis in which globular-stage embryos require polar auxin transport for cotyledon initiation (Jenik and Barton 2005). The under-developed condition of the orchid seed mimics this globular developmental stage, so it is not surprising that auxin redistribution in the protocorm would be a necessity for first leaf formation. Additional studies with the shoot meristem also emphasize the need for an appropriate distribution of auxin for leaf initiation. In *Arabidopsis*, the PIN-deficient shoot meristem requires exogenous IAA for lateral organ initiation and positioning (Reinhardt *et al.* 2000). Moreover, leaf initiation from tomato and maize shoot apices is inhibited by application of the PAT inhibitor, NPA (Reinhardt *et al.* 2000; Scanlon 2003).

Stunted shoot growth due to auxin application has been reported in other orchid species as well. In whole-seedling explant studies with *Phaphiopedilum* and *Coelogyne*, researchers found that shoot growth was inhibited when seedlings were cultured on media containing 2,4-D (Sharma and Tandon 1986; Wattanawikkit *et al.* 2011). A study by Hadley and Harvais (1968) reported a similar inhibition of seedling shoot growth when *Orchis* seeds were cultured on IAA-containing media. These authors also noted that protocorms were elongated in response to auxin, which may be the fleshy leaves of the auxin-treated 20 DAC seedlings described in the current work.

A second response seen in the young seedlings treated with auxins was an increase in protocorm diameters. Exogenous auxin application has also been found to enhance protocorm formation in other orchid species (Arditti 1967, 1979; Miyoshi and Mii 1995). A small increase in protocorm diameter was observed in the NPA-treated seedlings, but those treated with IAA and 2,4-D increased by 15–30 %. The weak NPA response and the lack of a response in TIBA- and monensin-treated seedlings may be due to a need for higher auxin levels coupled with uniform distribution for protocorm expansion.

Protocorm hair formation was increased several fold in auxin-treated 20 and 65 DAC seedlings, a response that is more indicative of root hairs than stem trichomes. Proposed functions for the protocorm hairs are a site for fungal association and absorption (Warcup 1985; Chang *et al.* 2005, respectively). Both of these functions are indicative of root hairs, but normal hair origin is the protocorm, a stem-like structure. Root hairs respond to auxin by increasing in length and number, but auxin does not promote trichome formation on stems and leaves, rather GA does (Rahman *et al.* 2002; Traw and Bergelson 2003; Ishida *et al.* 2008). In a study done with *Orchis* seedlings, it was reported that GA enhanced epidermal hair formation on the protocorm. However, in the same study, neither IAA nor cytokinins alone elicited this response, but some hair formation was observed when seed was cultured on media with IAA plus kinetin (Hadley and Harvais 1968). It is important to note that in the work of Hadley and Harvais (1968), seed was directly cultured on hormone-containing media, while in this study, early protocorms were sub-cultured at 10 DAC. Developmental timing of hormone exposure is critical to the type of response observed in the seedlings. Regardless, this dual response of protocorms to auxins and GA is intriguing, since it suggests that the protocorm may be both root and stem-like.

In this work, hairs of the older auxin-treated seedlings were numerous and randomly placed on the rhizome-like structures and fused, fleshy leaves. In contrast, the hairs of the 65 DAC control seedlings were restricted in ‘tufts’ on the distal end of the residual protocorm tissue/stem. In the younger seedlings (20 DAC), there was less of a response with the PAT inhibitors than in auxin-treated seedlings, which was consistent with the protocorm diameter data. Monensin and TIBA had a higher percentage of seedlings with hairs than the control (∼7-fold increase), but auxin promoted a 37- and 55-fold increase with IAA and 2,4-D, respectively. In addition, hair formation began at 15 DAC, which is well before the normal time in development (35 DAC) when hairs are appearing on the distal end of a majority of the seedlings under control conditions. This precocious formation, along with the random placement of hairs, rather than in distinct tufts, suggests that relatively high auxin levels in a specific location may be responsible for hair initiation.

To date, there have been no published studies that specifically compare orchid seedling hormone responses at multiple stages of seedling development. Researchers have reported whole-seedling explant responses to exogenously applied hormones, either grown from seed or sub-cultured at a single time point in development (Wotavova-Novotna *et al.* 2007; Sotthikul *et al.* 2010; Wattanawikkit *et al.* 2011). In this study, organ morphogenesis was significantly impacted by sub-culturing seedlings with auxin, and in some cases the response was seedling stage specific. As described above, first leaf formation at 20 DAC was dependent upon appropriate auxin delivery and/or concentration. The 145 DAC seedlings were exclusive in their formation of microshoots in response to auxins, and the generation of rhizome-like structures was unique to the auxin-treated 65 DAC seedlings. Consistent with their rhizome identity, these structures formed from nodes and leaf axils, and had a siphonostele tissue arrangement. Identification of these structures as rhizomes was further supported by the fact that auxin application has been shown to induce rhizome formation from leaf axils of *Cymbidium* (Shimasaki and Uemoto 1990). Further, rhizogenesis was reported to occur in callus tissue developed from young *Spathoglottis* seedling stems in response to auxins and varying the nutritional contents of the culture media (Bapat and Narayanaswamy 1977). The 65 DAC seedlings also formed PLBs, which were not observed in 20 DAC or 145 DAC seedlings. All of these plant structures, rhizomes, PLBs and microshoots, have been used successfully in propagation in *S. plicata* or other orchid species (Arditti 2008).

The overlapping auxin responses in the three seedling stages exhibited a reversion to non-specific, propagative tissue and, in older seedlings, leaf senescence. Adjacent time points, 20 and 65 DAC, shared the formation of fleshy leaves. In addition, callus tissue was commonly observed on fleshy leaves of 65 DAC seedlings that had appeared to fuse. Consistent with the fleshy leaves produced in PAT inhibitor- or auxin-treated 20 DAC seedlings, 18 % of the 65 DAC seedlings cultured with IAA or 2,4-D had fleshy leaves with hairs, unlike non-fleshy leaves and the leaves of control seedlings that completely lacked hairs. These results suggest that auxins may be converting the 65 DAC seedling leaves into protocorm-like structures with hairs. Callus formation and leaf necrosis were shared responses in the 65 and 145 DAC seedlings. Moreover, the unique propagative structures of 65 and 145 DAC seedlings, rhizome-like structures and microshoots, respectively, were induced from leaf axils as normal seedling leaves became necrotic. Auxin has been shown to promote ethylene production in orchids (Zhang and O'Neill 1993; Peres *et al.* 1999). Older seedling leaves may be sensitive to this hormone, causing leaf necrosis. Overall, auxins seem to be shutting down normal growth patterns in older seedlings and initiating non-specific tissue growth that is more indicative of early development and/or vegetative propagation.

## Conclusion and forward look

This work was the first to characterize auxin responses in two important events of orchid seedling development: first leaf formation and the induction of protocorm trichomes. In future work, researchers can use this knowledge to begin studies on molecular events coordinated with orchid germination and seedling establishment. The auxin-controlled first leaf initiation from protocorms mirrors events at the apical meristem and those in typical angiosperm embryogenesis. Therefore, orchid seed culture may provide a simple system for future studies in leaf initiation, whether from the meristem or in embryos. Similarly, hair induction is easily promoted by auxin application; thus researchers who study hair formation may find this system attractive for elucidating pathways of epidermal cell differentiation. Moreover, this work addressed the role of auxin in forming structures for propagation, which are necessary for future conservation efforts and current horticultural needs.

## Sources of funding

This work was supported by the US Department of Education, Title V STEM (award number P031C080019) and the USDA (NIFA award number 2011-38422-30942).

## Contributions by the authors

S.D.N. planned the research, and G.A.W. conducted initial trials, collected data and performed initial analysis. S.D.N. performed additional trials and collected data for final analysis. S.D.N. wrote the manuscript and G.A.W. contributed to the discussion of the work and the review of the manuscript.

## Conflict of interest statement

None declared.
